# Er:YAG laser, piezosurgery, and surgical drill for bone decortication during orthodontic mini-implant insertion: primary stability analysis—an animal study

**DOI:** 10.1007/s10103-017-2381-9

**Published:** 2017-11-10

**Authors:** Jacek Matys, Rafał Flieger, Gianluca Tenore, Kinga Grzech-Leśniak, Umberto Romeo, Marzena Dominiak

**Affiliations:** 1 “Sapienza” University of Rome, Rome, Italy; 2 Private Dental Healthcare, Lipowa 18, 67-400 Wschowa, Poland; 3 Private Dental Healthcare, Kościan, Poland; 4 Department of Oral and Maxillofacial Sciences, “Sapienza” University of Rome, Rome, Italy; 5 Dental Surgery Department, Medical University of Wroclaw, Wroclaw, Poland

**Keywords:** Er:YAG laser, Orthodontic mini-implants, Periotest, Piezosurgery, Primary stability

## Abstract

It is important to identify factors that affect primary stability of orthodontic mini-implants because it determines the success of treatment. We assessed mini-implant primary stability (initial mechanical engagement with the bone) placed in pig jaws. We also assessed mini-implant insertion failure rate (mini-implant fracture, mini-implants to root contact). A total of 80 taper-shaped mini-implants (Absoanchor® Model SH1312-6; Dentos Inc., Daegu, Korea) 6 mm long with a diameter of 1.1 mm were used. Bone decortication was made before mini-implant insertion by means of three different methods: Group G1: Er:YAG laser (LiteTouch®, Light Instruments, Yokneam, Israel) at energy of 300 mJ, frequency 25 Hz, fluence 38.2 J/cm2, cooling 14 ml/min, tip 1.0 × 17 mm, distance 1 mm, time of irradiation 6 s; Group G2: drill (Hager & Meisinger GmbH*,* Hansemannstr, Germany); Group G3: piezosurgery (Piezotom Solo, Acteon, NJ, USA). In G4 group (control), mini-implants were driven by a self-drilling method. The primary stability of mini-implants was assessed by measuring damping characteristics between the implant and the tapping head of Periotest device (Gulden-Medizinteknik, Eschenweg, Modautal, Germany). The results in range between − 8 to + 9 allowed immediate loading. Significantly lower Periotest value was found in the control group (mean 0.59 ± 1.57, 95% CI 0.7, 2.4) as compared with Er:YAG laser (mean 4.44 ± 1.64, 95% CI 3.6, 5.3), piezosurgery (mean 17.92 ± 2.73, 95% CI 16.5, 19.3), and a drill (mean 5.91 ± 1.52, 95% CI 5.2, 6.6) (*p* < 0.05). The highest failure rate (33.3%) during mini-implant insertion was noted for self-drilling method (G4) as compared with G1, G2, and G3 groups (*p* < 0.05). The small diameter decortication by Er:YAG laser appeared to provide better primary stability as compared to drill and piezosurgery. Decortication of the cortical bone before mini-implant insertion resulted in reduced risk of implant fracture or injury of adjacent teeth. The high initial stability with a smaller diameter of the mini-implant resulted in increased risk of fracture, especially for a self-drilling method.

## Introduction

Orthodontic mini-implants, as a type of anchorage system, are becoming more popular in modern orthodontic treatment [1]. Anchorage instability associated with classical anchorage process was eliminated by using mini-implants [2]. However, according to Beak et al. [3], mini-implant breakdown occurs in short time following early force loading. Therefore, improving early-phase stability is crucial for enhancing the reliability of mini-implant treatment [4].

Decortication refers to the removal of the cortical portion of the alveolar bone. Clinically, this procedure may be performed by using various cutting devices such as drills, piezosurgery, or appropriate lasers. Then, orthodontic implants are inserted by hand or mechanical tools into the prepared cortical bone. In specific cases (low bone density), the orthodontic implant is driven without decortication by a self-drilling method. An appropriate method of implant insertion is a key factor to achieve good primary stability, sufficient to the immediate loading of the orthodontic mini-implants [5].

Determining primary stability after insertion may improve success predictability. Measurement of insertion torque is considered as one of the most comprehensive methods used today. Stability of mini-implants has variable value during the healing phase due to the bone remodeling [6]. It could be useful to assess the stability of mini-implants in each phase of orthodontic treatment to evaluate an optimal loading protocol. Literature review provides an account of various methods of measuring implant stability [7].

One of the most popular devices for evaluating the primary stability is Periotest (Medzintechnik Gulden e K, Modautal, Germany). In this device, a small pistil is accelerated toward the implant, which is deflected depending on its peri-implant situation [8]. A numerical scale ranging from − 8 to + 50 was used to evaluate the damping characteristics of the peri-implant tissue where the lower values indicate higher implant stability [9].

Mini-implant primary stability depends on various factors. Marquezan et al. [10] in their study showed that the bone cortical thickness influences mini-implant primary stability. Therefore, before mini-implant insertion, the bone density needs to be evaluated. An efficient method of bone density assessment using Hounsfield analysis by computer tomography examination was performed by Carl Misch in 1999 [11]. The Misch Classification ranges from a dense, cortical bone to immature, non-mineralize bone (D1 > 1250 HU, D2 850–1250 HU, D3 350–850 HU, D4 150–350 HU, D5 < 150 HU) [11].

Many authors [12–14] emphasized that the primary stability of orthodontic mini-implants is affected not only by the bone quality but also by their length, diameter, and design characteristics. Furthermore, Yi Lin et al. [15] indicated that both patient and mini-implant-related factors affected the primary stability and long-term therapeutic success. Novsak et al. [16] considered that the way of inserting mini-implants also has an impact on primary stability.

Various bone cutting devices could have an influence on the implant primary stability. In the last two decades, the erbium family of laser wavelength have gained more and more popularity. These lasers operate at a wavelength of 2.78 (Er,Cr:YSGG) and 2.94 (Er:YAG) 휇m and show good absorption in water in the infrared range of the electromagnetic spectrum. Er:YAG laser with the highest absorbance coefficient in the water allows to perform the surgery in both soft and hard (bone, tooth) tissues with maximal effectiveness. Moreover, the beam of Er:YAG laser is absorbed in the superficial layer of tissue and does not penetrate or scatter more than several microns [17, 18].

The aim of the study was to examine and compare the mini-implant primary stability placed in pigs mandible by a manual method: without and with bone decortication using drill, piezosurgery, and erbium:yttrium-aluminum-garnet laser by means of Periotest device. We also assessed mini-implant insertion failure rate (implant fracture, implant to root contact) and carbonization occurrence in the soft tissue during Er:YAG irradiance.

## Materials and methods

Forty mandibles of 10-month-old male pigs were used in this study. Three different devices for cortical bone osteotomy were applied before the orthodontic mini-implant insertion.

### Sample preparation

The specimens (*n* = 80) have been prepared by cutting each mandible in anterior incisors region. The specimens were randomly divided into four groups (*n* = 20), then were washed with the tap water, and left for 4 h before the research was commenced. The approval of the Local Ethics Committee of Wrocław Medical University, Faculty of Dentistry, was obtained (KB-527/2016).

### Surgical procedure

**Fig. 1:**
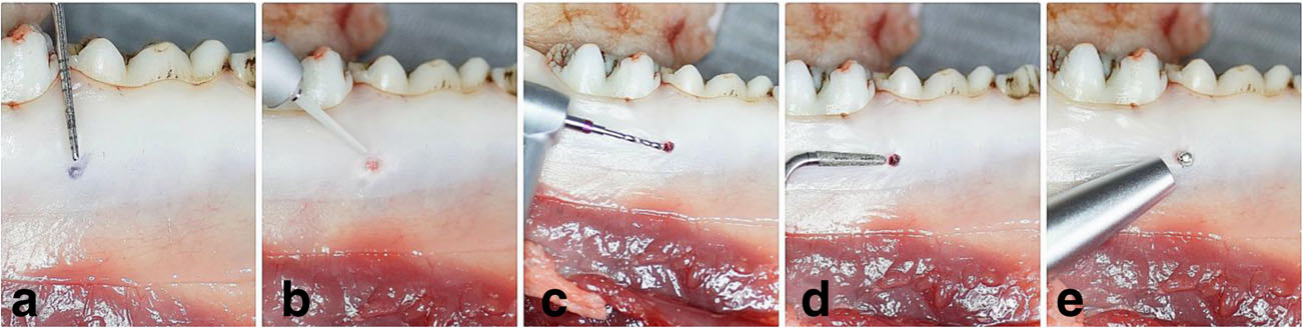
Decortication of the bone in the area between forth premolar (P4) and first molar (M1) for mini-implant insertion. **a** Marked position of the MI. **b** Er:YAG laser tip. **c** A surgical drill. **d** Piezosurgery tip. **e** Measuring PTV by Periotest device

In the study area between fourth premolar (P4) and first molar (M1) of the mandible, the bone decortication was performed before mini-implant insertion by means of Er:YAG laser (*n* = 20), surgical drill (*n* = 20), and piezosurgery (*n* = 20). The remaining specimens (*n* = 20) were left without bone decortication and served as a control group. A total number of 80 taper-shaped mini-implants (Absoanchor® Model SH1312-6; Dentos Inc., Daegu, Korea), made of titanium alloy, 6 mm long with a diameter of 1.1 mm, were placed at the angle of 90 degrees by hand tool into the prepared cortical bone or were driven without decortication by self-drilling method. The bone decortication was evaluated by placing the periodontal probe into the prepared orifice (Fig. 1).

### Measurement procedure

The primary stability of the mini-implants was measured by means of Periotest device (Gulden-Medizinteknik, Eschenweg, Modautal, Germany), and the results of Periotest value (PTV) between the four groups were statistically compared.

The position of inserted mini-implants was controlled by cone-beam computed tomography (CBCT) examination (Kodak 9000 3D, Carestream/Trophy, Marne-la-Vallée, France).

**Fig. 2:**
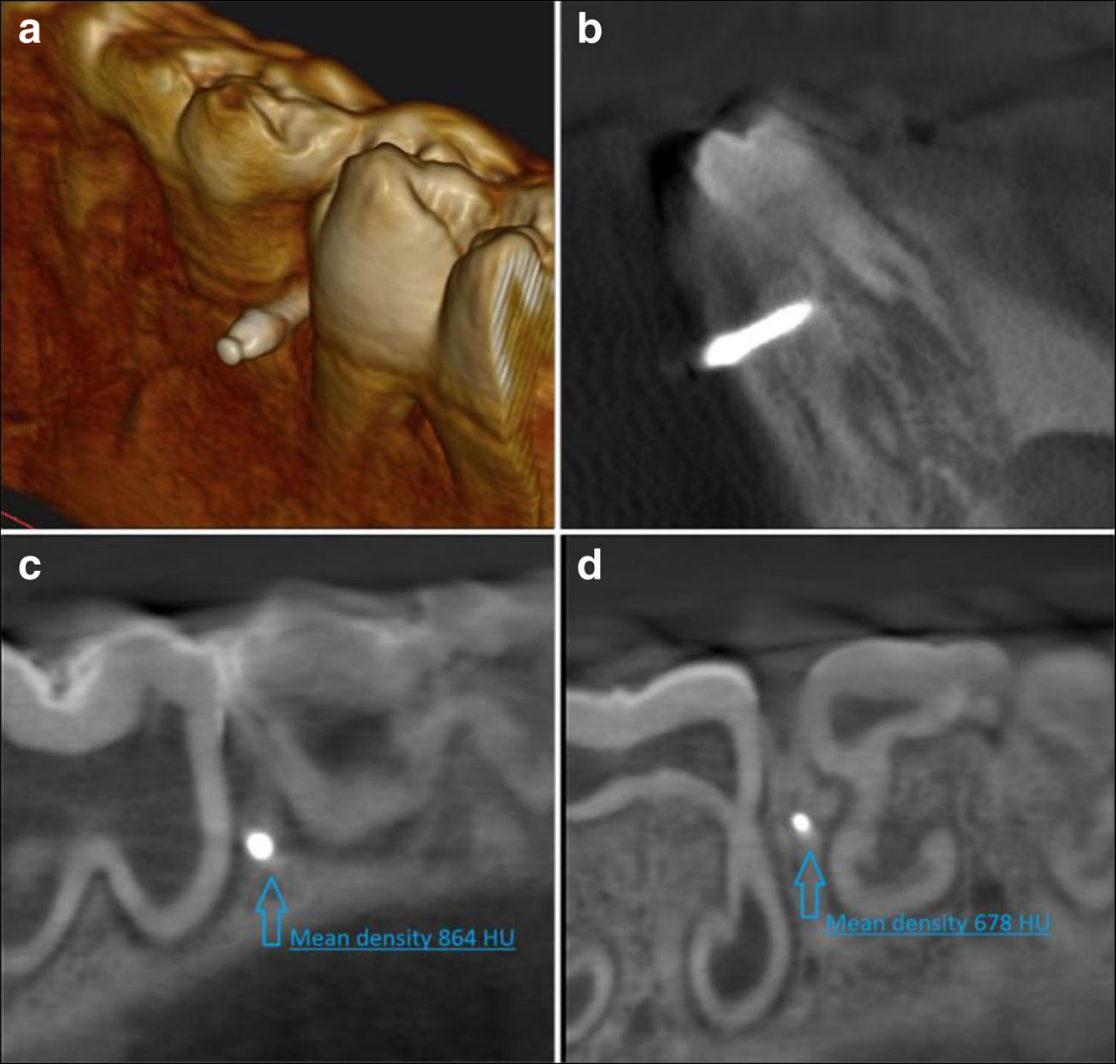
Assessment of the mini-implant position in the mandible using cone-beam computed tomography (CBCT). **a** 3D image of the mini-implant after insertion. **b** A frontal plane of the mini-implant placed in the pig mandible. **c** A bone density at implant’s collar level in HU units. **d** A bone density at implant’s apex level in HU units

Bone density at the level of first (collar) and last (apex) of mini-implant thread was evaluated by means of the Hounsfield unit (HU) analysis. The measurements were taken using a CBCT software (SimPlant 14, Materialize, Belgium) (Fig. 2).

CBCT analysis was performed to include in the study only the samples with a similar bone quality at implant collar and apex to minimize the influence of different bone density on the implant primary stability.

The exclusion criteria of the study were set as follows: bone density different from D2 (at collar level) and D3 (at apex level) of the mini-implants, mini-implant fracture during insertion, and the gap between mini-implant and tooth was less than 0.5 mm.

### Study groups

The study specimens (*n* = 80) were divided into four groups: G1 (*n* = 20), G2 (*n* = 20), G3 (*n* = 20), and G4 (*n* = 20).

G1 group: Er:YAG laser (LiteTouch®, Syneron Dental, Yokneam, Israel) in operation mode for hard tissues (HT) was used with the following parameters: energy 300 mJ, frequency 25 Hz, energy density per pulse 38.2 J/cm2, water spray cooling 14 ml/min, tip angle set at 90°, size of the tip 1.0 × 17 mm, distance 1 mm, and time of irradiation 6 s.

G2 group: a drill 1.0 mm in diameter (Hager & Meisinger GmbH, Hansemannstr, Germany) in a high-speed contra-angle handpiece (Intra C09-C3 27:1 Kavo, Biberach, Germany) was operated with a physiodispenser (Intrasurg300®, Kavo, Biberach, Germany), at speed of 1000 rpm and with water spray for cooling 20 ml/min.

G3 group: Piezosurgery unit (Piezotom Solo, Acteon, NJ, USA) was used with the following parameters: tip TKW1, diameter 1.35 mm, power D1, and water spray cooling 20 ml/min.

G4 group (control): no decortication before mini-implant insertion.

### Statistical analysis

The statistical analysis was based on one-way ANOVA variance analysis conducted using Statistica 10 software (StatSoft, Krakow, Poland). Pair comparisons were carried out based on Tukey test at significance levels *p* = 0.05. To evaluate the mini-implant failure frequency, the chi-square RxC test was carried out. The final number of variables in each group was not equal in due accordance to the exclusion criteria (failed cases). Values below *p* = 0.05 were considered to be statistically significant.

## Results

The lowest mean PTV (0.59) was measured in control group (G4) for self-drilling mini-implants as compared to experimental groups (*p* = 0.0002) (Table 1).

**Table 1:** The mean Periotest values (PTV) and standard deviation (SD) in the study groups

Variable	Mean PTV ± SD	95% CI	PTV range	*p* value
Group 1 (*n* = 18)	4.44 ± 1.64	3.6–5.3	1.7–6.8	ANOVA analysis, *p* < 0.05 G1 vs. G2, *p* = 0.0983 G1 vs. G3, *p* = 0.0002 G1 vs. G4, *p* = 0.0002 G2 vs.G3, *p* = 0.0002 G2 vs.G4, *p* = 0.0002 G3 vs.G4, *p* = 0.0002
Group 2 (*n* = 19)	5.91 ± 1.52	5.2–6.6	3.6–9.2	
Group 3 (*n* = 17)	17.92 ± 2.73	16.5–19.3	12.6–23	
Group 4 (*n* = 15)	0.59 ± 1.57	0.7–2.4	−2.6 - 2.3	

*95% CI* confidence interval

**Fig. 3:**
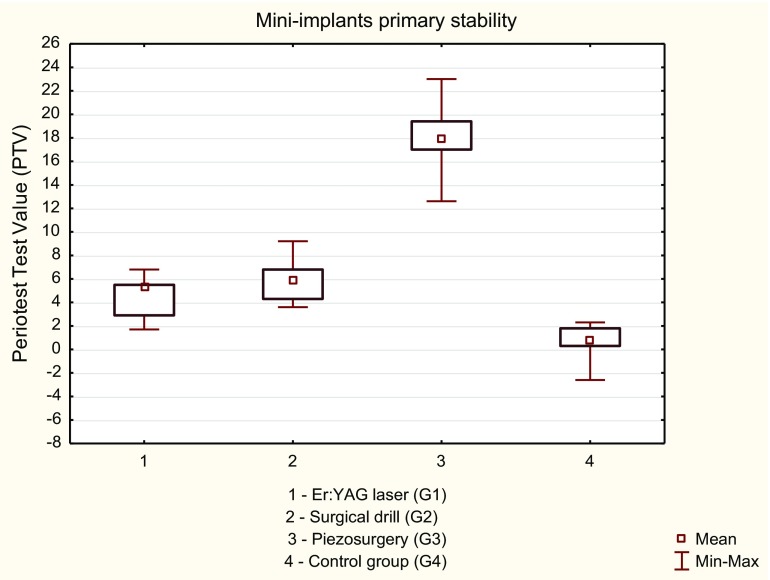
The analysis of the mini-implant primary stability conjugated with the PTV after bone decortication by means of Er:YAG laser, drill, and piezosurgery

The analysis of the mini-implant primary stability conjugated with PTV after bone decortication revealed significant higher primary stability (lower PTV) for specimens prepared using Er:YAG laser (G1) in comparison with piezosurgery (G3) (*p* = 0.0002). There were no significant differences between the Er:YAG laser and surgical drill (G2) (*p* = 0.0983) (Fig. 3).

**Fig. 4:**
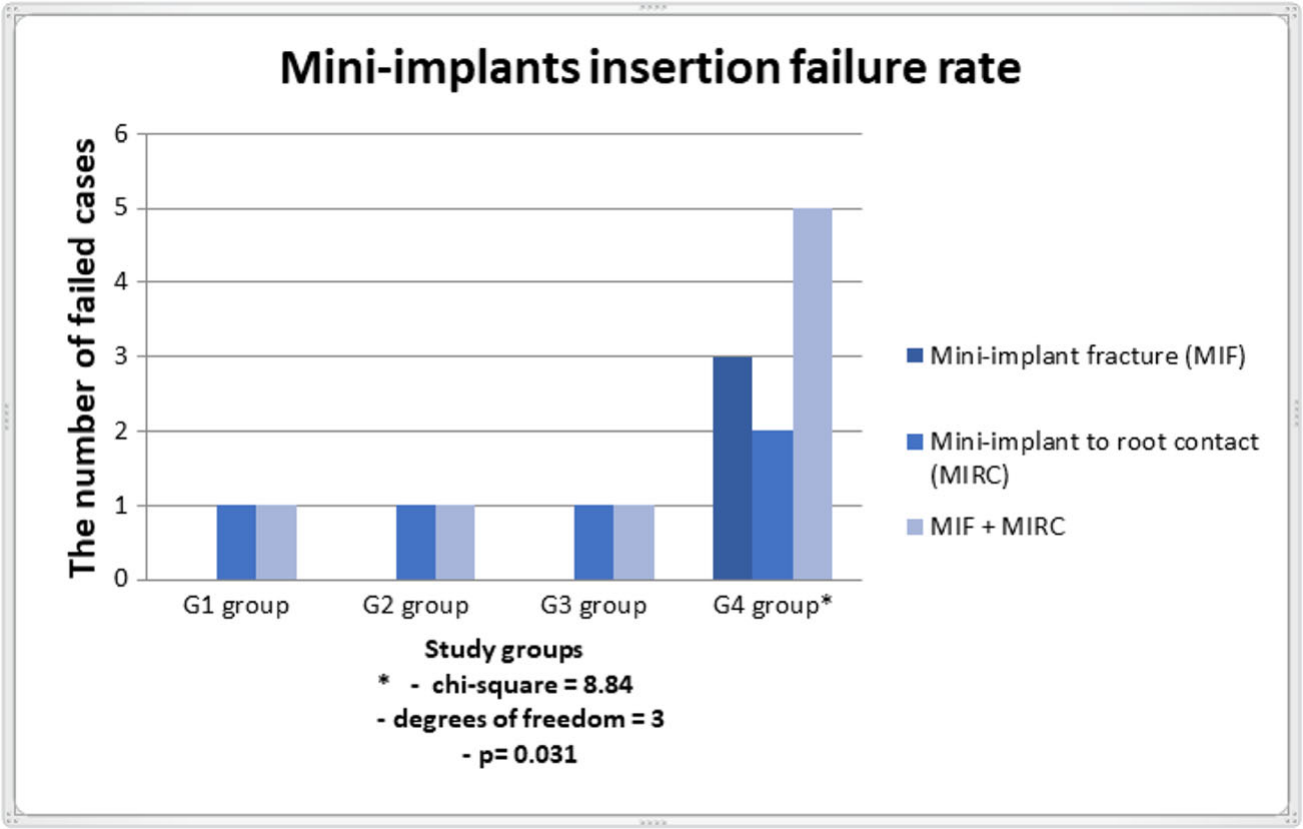
The failure rate for mini-implants inserted without bone preparation in control group (G4) in comparison with decorticated specimens (G1, G2, G3)

The failure rate for mini-implants inserted without bone preparation in control group (G4) was significantly greater (33.3%) in comparison with decorticated specimens (G1, G2, G3) (*p* = 0.031) (Fig. 4).

## Discussion

Literature review provides no record of comparative ex vivo or in vivo studies which evaluate the use of rotary instruments, Er:YAG laser and piezosurgery in the procedures of orthodontic mini-implant placement. In our study, we used a CBCT scanner to control the degree of accuracy of the mini-implant position. Furthermore, the use of CBCT allows for precise evaluation of bone density, which has a significant impact on primary stability and predictability of this procedure. The specimens included in this study at the cortical bone and 6 mm deeper had the bone density of D2 and D3 according to Misch scale. Three-dimensional digital radiographs allow to provide similar density of the specimens in which mini-implants were driven. Furthermore, CBCT radiogram allows to examine the proper mini-implant placement between roots, especially in areas with difficult anatomy [19].

The two important factors, which influence primary stability of mini-implants, are dimension and cortical bone thickness [20]. Therefore, the assessment of bone density is crucial before mini-implant insertion. All mini-implants used in our study were equal in diameter and length. For all mini-implants utilized in the statistical analysis, the bone density at the mini-implant collar or apex were D2 or D3, respectively. The results of our study clearly indicated that the primary stability of the mini-implants was affected not only by their type as was pointed in different studies [22, 23] but also by the surgical procedure and the use of surgical devices such as Er:YAG laser or piezosurgery.

Marquezan et al. [10] and Motoyoshi et al. [21] proved that there is a positive association between mini-implant primary stability and cortical thickness (CtTh) of the receptor site. In our study, the cortical bone density (at first mini-implant thread) was D2 (850-1250 HU), which corresponds to the CtTh level in Marquezan’s study. Our surgical procedure aimed at making decortication point of the cortical lamellae using Er:YAG laser, drill, and piezosurgery before placing the mini-implants. The highest primary stability after initial decortication was reached with the Er:YAG laser at 300 mJ and 25 Hz. The use of this laser allowed to prepare the cortical bone by lasing all specimens for 6 s. In our opinion for specimens with density in a range of D1-D2, the preparation of bone is the key factor to avoid failures when placing mini-implants. Thus, in the experimental groups, the specimens were prepared in the area of cortical bone only superficially without preparing a tunnel for the whole length of the mini-implants. This technique allowed to insert the implant with low PTV score (high primary stability) and resulted in decreasing the risk of placing the mini-implants at wrong angle when too much force is applied during implantation in dense bone.

The results of this study showed a mean PTV score of 4.44, 5.91, 17.92, and 0.59 for Er:YAG laser, drill, piezosurgery, and control (self-drilling), respectively. It was proven that density of the bone, cortical thickness as well as the type of the micro-screw, affected the mini-implant primary stability [22–24]. We found a positive correlation between cortical orifice’s diameter and the mini-implant primary stability in all the groups. Preparation of cortical bone using Er:YAG laser resulted in a less homogeneous opening as compared to drill. This resulted in increased contact coefficient between bone and implant, which consequently promoted the mini-implant primary stability. On the other hand, the smallest tip available for piezosurgery device utilized in this study has the diameter of 1.35 mm which resulted in a higher PTV score (lower primary stability). Better results (higher primary stability) using piezosurgery system applied in this study could have been achieved by utilizing tip with diameter lower than 1.35 mm. Unfortunately, there are no special tips on the piezosurgery handpiece designed for the orthodontic mini-implant insertion currently available in the market. Higher primary stability in the present study was obtained for mini-implants, which were driven without bone decortication which could have been triggered by the highest bone-contact value of the specimens from control group. Dilek et al. [25] emphasized the importance of primary stability at an early load of the mini-implants. They concluded that mini-implants, which are especially designed for immediate loading, can only be loaded immediately if their Periotest values are between the range of − 8 to + 9. In our study, Periotest value within this range was observed in three of four study groups: G4 (self-drilling), G1 (Er:YAG laser), and G2 (drill). Our results indicated that each of these methods can be used in orthodontic treatment based on immediately loading of mini-implants.

We would like to highlight that our present work was an ex vivo study with all typical limitations, for example, a different chemical composition and the biological properties of the “ex vivo” specimens as compared with “in vivo” tissue, mainly because of the absence of the blood circulation and greater cortical thickness. The absence of blood circulation could account for higher temperature rise in the animal “ex vivo” model. In our present study, we did not found carbonization in tested samples by means of visual inspection. Romeo et al. [26] in their research observed by means of an optical microscope poor peripheral carbonization of the bone after lasing with Er:YAG device. Despite minor thermal damage accompanying decortication process, the use of Er:YAG laser seems to be safe and efficient in bone surgery. [27, 28] Another important limitation found in this study is greater cortical thickness of the pig mandibles. Therefore, we decided to include in the research only the samples with a similar density at cortical region (D2) and at mini-implant apex area (D3). These types of the bone dominate mainly in the mandible or are common for both anterior and posterior jaw regions [29]. Our findings should be confirmed in the human “in vivo” model as well; however, the results of this research indicate that clinicians working with the mini-implants in their practice should achieve similar results in the primary stability in aforementioned regions of the jaw when using the protocol described in this study. Furthermore, another limitation of this study is measurement error conjugated with inaccurateness of measuring devices. That way, these findings should be confirmed by histological analysis concerning a bone to implant contact (BIC) in animal “in vivo” model.

The present research recorded the failure rate during mini-implant insertion. We found in three cases fractures of mini-implants between the head and first thread for implants placed without primary decortication. Despite the highest mean values of primary stability as indicated by Periotest obtained in the control group, it is recommended to perform decortication of cortical lamina in order to avoid complications. Furthermore, in the group without bone decortication, two mini-implants touched the root of the adjacent tooth. This could mean that the use of excessive force during implant insertion with no bone decortication may disturb the correct trajectory of the implant. The mini-implant insertion in a dense bone determines the high initial mechanical engagement between the bone and implant. This could induce the risk of implant position displacement over the bone wall during initial contact of the mini-implant with a cortical lamina. We concluded that the cortical bone decortication prevents injury of adjacent teeth.

We found that the high initial stability measured by Periotest (mean 0.59 ± 1.57, 95% CI 0.7, 2.4) when placing the mini-implants without decortication could be conjuncted with the implant fracture. We concluded that the high initial stability and a smaller diameter of the mini-implant result in increased risk of fracture especially in the self-drilling method. It has been demonstrated that the insertion torque of the tapered mini-implants placed in a maxilla and mandible was significantly higher than that of the cylindrical. [23] We used taper-shape mini-implants, which may also have an impact on the possible fracture of mini-implants. A similar conclusion regarding higher torque in the tapered mini-implants can be found in many other scientific reports. [30–32] We recommend to perforate cortical lamina of the bone with a density over 840 HU to decrease the forces needed to insert implant with minimal PTV’s values needed to anchorage the orthodontic appliance. This protocol allows immediate loading of mini-implants and decreases the risk of injury of the root especially in narrow areas, e.g., between labial roots of the molar. However, additional studies using the erbium laser with the histological analysis of a mini-implant to bone contact value are needed to confirm the results of this study in animal and human in vivo models. Also, the results of primary stability should be checked with other implant diameter size, especially for diameters greater than 1.1 mm.

## Conclusion

The small diameter decortication by Er:YAG laser appeared to provide better primary stability as compared to drill and piezosurgery. Decortication of the cortical bone before mini-implant insertion results in less risk of implant fracture or injury of adjacent teeth. High initial stability with a smaller diameter of the mini-implant result in increased risk of its fracture especially in the self-drilling method. Bone decortication by means of Er:YAG laser or drill provides sufficient value of primary stability for immediate loading protocol. The Er:YAG laser could be utilized as an alternative to surgical drills during insertion of mini-implants in orthodontic treatment.
